# Laser tracheobronchoplasty: a novel technique for the treatment of symptomatic tracheobronchomalacia

**DOI:** 10.1007/s00405-016-4349-y

**Published:** 2016-10-20

**Authors:** Paul Castellanos, Manjunath MK, Ihab Atallah

**Affiliations:** 1 Department of Otolaryngology, University of Alabama at Birmingham, Boshell Building 563, Birmingham, AL 35233 USA; 2 Colombia Asia Referral Hospital, Yeshwanthpur, Bangalore, India; 3 Otolaryngology-Head & Neck Surgery Department, Grenoble University Hospital, BP 217, 38043 Grenoble Cedex 09, France

**Keywords:** Tracheobronchomalacia, Holmium laser, Tracheobronchoplasty, Wheezing, Cough, Respiratory failure, Excessive Dynamic Airway Collapse (EDAC)

## Abstract

The management of tracheobronchomalacia is a very challenging problem with few treatment options. This study aims to evaluate the outcomes of a novel surgical treatment for membranous tracheobronchomalacia. A consecutive series of patients with tracheobronchomalacia were treated with two to three holmium laser scarring surgeries of the hyperdynamic tracheal and bronchial walls for the purpose of stiffening them through fibrosis. Patients filled out a Dyspnea Index questionnaire before and after treatment. Ten patients were treated for their tracheobronchomalacia with a mean age of 54 years. Symptoms included severe dyspnea, dry cough, recurrent pulmonary infections, and respiratory failure. Fifty percent of patients presented with wheezing refractory to traditional treatment. Tracheobronchomalacia was associated with gastroesophageal reflux disease (*n* = 8), obstructive sleep apnea (*n* = 5), and tracheal stenosis (*n* = 3). Only 50 % of patients presented with morbid obesity. All cases showed significant improvement of their respiratory symptoms with a mean postoperative difference of 22.3 out of a maximum impairment score of 40 (*P* < 0.01) on the Dyspnea Index. The mean number of procedures was 2.3 per patient with the average laser energy delivered per procedure of 1600 J. Laser tracheobronchoplasty is a safe, easy to adopt, and effective technique for the treatment of membranous tracheobronchomalacia. It presents a simple alternative to the commonly used procedures like endoluminal stenting and open tracheobronchoplasty.

**Electronic supplementary material:**

The online version of this article (doi:10.1007/s00405-016-4349-y) contains supplementary material, which is available to authorized users.

## Introduction

Tracheomalacia is considered the diffuse or segmental weakness and increased collapsibility of the trachea. Tracheobronchomalacia (TBM) refers to tracheomalacia that extends into the mainstem bronchi and even the lobar and segmental bronchi. Patients with airway malacia typically present with nonspecific chronic respiratory complaints, including dyspnea, cough, and recurrent infections that do not respond to traditional treatments such as bronchodilators or steroids [1].

TBM may be underdiagnosed because of the presentation with a variety of non-specific symptoms similar to patients with asthma and chronic obstructive pulmonary disease (COPD). TBM can cause progressive pulmonary disease which may lead to respiratory failure and death. Currently, there is controversy with regards to the difference between normal expiratory airway collapse, excessive dynamic airway collapse (EDAC) and TBM. Some authors consider TBM the weakening of the airway cartilage and EDAC as the result of posterior wall redundancy and weakening [2]. However, for practical purposes, the focus of the treatment of TBM and EDAC is clinically the same [3].

There are congenital and acquired forms of TBM. Congenital TBM can be an isolated finding in otherwise healthy infants, but it is more commonly seen in premature infants [4]. Acquired TBM occurs secondary to indwelling tracheostomy and endobronchial tubes, chest trauma, chronic tracheobronchitis, pulmonary resection, tracheal malignancy, and inflammation like relapsing polychondritis [5]. Acquired idiopathic TBM has been reported in cases with no known history of intubation or other tracheobronchial insult [6, 7].

There are no well-defined guidelines for diagnosing or treating TBM. Positive-pressure ventilation (CPAP, BiPAP, or PEEP) can be helpful, especially in an acute setting, but it is not curative [8–10]. Tracheostomy may stent the malacic airway and can provide invasive ventilatory support when necessary. In cases of focal cervical tracheomalacia, the tracheostomy tube alone may be effective but only if it bypasses the affected malacic segment. Tracheostomy, however, can be complicated by secondary tracheomalacia and stenosis at the stoma site. In addition, from a physiological standpoint, tracheostomy may exacerbate diffuse TBM because it bypasses the physiological function of the glottis to maintain positive transmural pressure on exhalation that keeps the airway lumen patent. Tracheostomy should not be considered a first line treatment in most cases [11]. Endoluminal stent placement is a commonly used treatment option with many drawbacks [2, 3, 7, 8, 10]. Other surgical treatment alternatives are laryngotracheal reconstruction with grafts or external tracheal supports, tracheal resection with a tightening of the posterior wall, and trans-thoracotomy tracheobronchoplasty using synthetic biocompatible meshes to strengthen the redundant posterior wall to prevent expiratory collapse into the airway; all of these treatment options may provide initial improvement in symptoms [2, 9]. However, they present a high rate of complications, including fatality, and cannot be considered as the treatment of choice for TBM. Unfortunately, one of the most common treatments for patients who have TBM is chronic prednisone therapy, which is known to do little to improve ventilatory dynamics in this population and renders the patient steroid dependent, Cushinoid, and often morbidly obese [12–14].

Diagnostic and therapeutic bronchoscopic procedures in pulmonology have been applied for a more selective and tailored approach to reduce patients’ morbidity and mortality. Laser is one of the tools used to endoscopically treat various pathologies of the tracheobronchial tree [11]. The purpose of this study is to evaluate the outcomes of a novel, endoscopic surgical approach using laser for the treatment of this challenging respiratory disease.

## Materials and methods

### Patient selection and preoperative evaluation

A cohort of all adult patients undergoing surgical intervention for TBM was retrospectively reviewed at a tertiary referral Laryngology and Bronchoesophagology Center. Only patients who underwent an endoscopic laser tracheobronchoplasty for primary TBM were included. Comorbid conditions that were included were tracheal stenosis, obstructive sleep apnea (OSA), and morbid obesity. Patients with conditions such as Mounier-Kuhn, relapsing polychondritis, tracheobronchopathia osteochondroplastica or had an existing tracheostomy were excluded as either very rare or warranting description in a separate report.

Ten patients were included in this study. There were two men and eight women with a mean age of 54 years (range 29–75 years). Their main symptoms included severe dyspnea, dry cough, recurrent bacterial and fungal bronchitis, and respiratory failure. Fifty percent of patients presented with wheezing refractory to corticosteroid and bronchodilator therapy. TBM was associated with gastroesophageal reflux disease (GERD) (*n* = 8), OSA (*n* = 5), and tracheal stenosis (*n* = 3). Five patients presented with morbid obesity. Two of ten patients had a prior tracheostomy but had already been decannulated 6–9 months before performing laser surgery. All the patients in this series were either on high dose prednisone on presentation or had a history of repeated steroid courses that never definitely altered their symptom profile.

After a comprehensive history and physical examination was performed, all patients underwent a sedation-free, trans-nasal, flexible bronchoscopy under local anesthesia; a topical solution of 4 % lidocaine hydrochloride was instilled through the instrument channel. A color high resolution video, flexible bronchoscope (KayPENTAX, New Jersey, USA) with a 5.1 mm outer diameter was used to make or confirm the diagnosis of TBM and to determine its severity. Endoscopy was performed with the patient in a sitting position. All patients were instructed to breathe naturally, then to take a deep breath, and exhale fully. Videos were recorded through the end of forced exhalation at the following sites: proximal trachea, mid-trachea and distal trachea proximal to the carina, right and left mainstem bronchi, and lobar and segmental bronchi. The maneuver was repeated at least twice to ensure that maximal airway collapse during exhalation was visualized. Examination was also done by asking the patient to cough. Coughing produces high exhalation velocities that can unmask occult airway collapsibility. At the end of the exam, the topography of airway collapse greater than 50 % was identified. In addition to the dynamic, flexible bronchoscopy study, all patients underwent inspiratory and end-expiratory chest CT scans to detect changes in tracheal sagittal diameters between inspiration and the end of expiration. All patients underwent pulmonary function tests with flow-volume loops and filled out a Dyspnea Index questionnaire (score range: 0 as asymptomatic to 40 with severe symptoms) preoperatively and 12 weeks postoperatively [15]. The preoperative Dyspnea Index Questionnaire showed a mean score of 35.7, range of 32–40, indicating severe dyspnea.

On auscultation on our initial exam, six patients presented with loud polyphonic wheezing associated with a rhonchorous coughing in five of these cases. All had a history of course wheezing on referring examination. On pulmonary function tests, five cases showed obstructive lung disease, two cases had a mixed restrictive-obstructive defect, and four cases were considered within normal limits. In eight cases, there was no significant change in the flow-volume loop after administration of bronchodilators. Only three patients had a significant airway collapse (>50 % change) detected on the dynamic CT scan of the chest.

### Laser tracheobronchoplasty

Patients diagnosed with TBM underwent laser tracheobronchoplasty performed by the senior author (PFC) or his fellows under his direction (IA and MMK). Informed consent was obtained from all patients included in the study. All procedures performed in the study were in accordance with the ethical standards of the institution and with the 1964 Helsinki declaration and its later amendments.

**Fig. 1:**
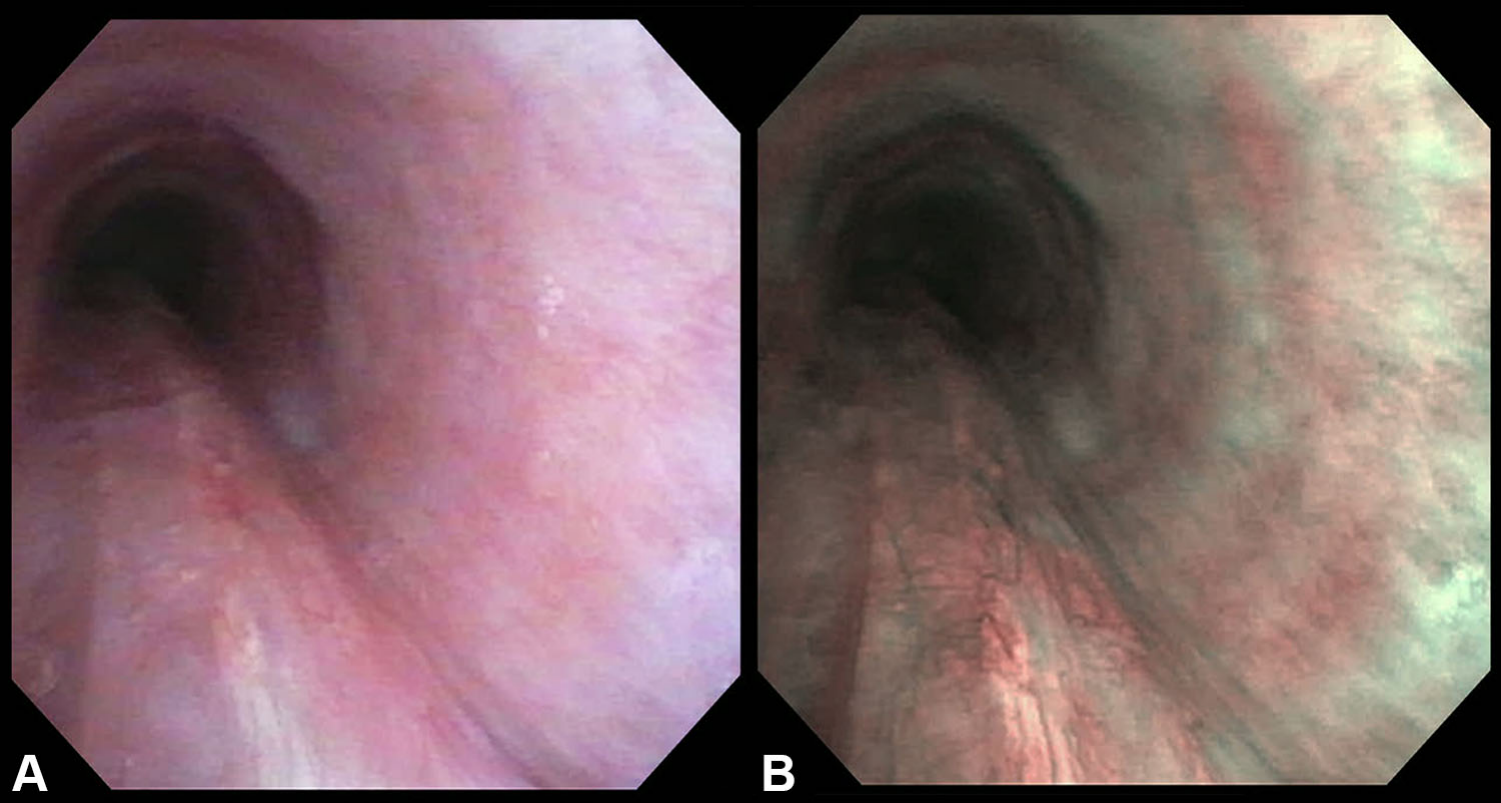
This pair of figures shows the same distal tracheal mucosa. **a** The mucosa under regular light shows a vague scar pattern. **b** The mucosa viewed with narrow band imaging (NBI, Olympus EXERA III system, Tokyo, Japan) that accentuates the visualization of the scar pattern. The longitudinal scars are clearest; this is consistent with these laser movements being typically the deepest

**Fig. 2:**
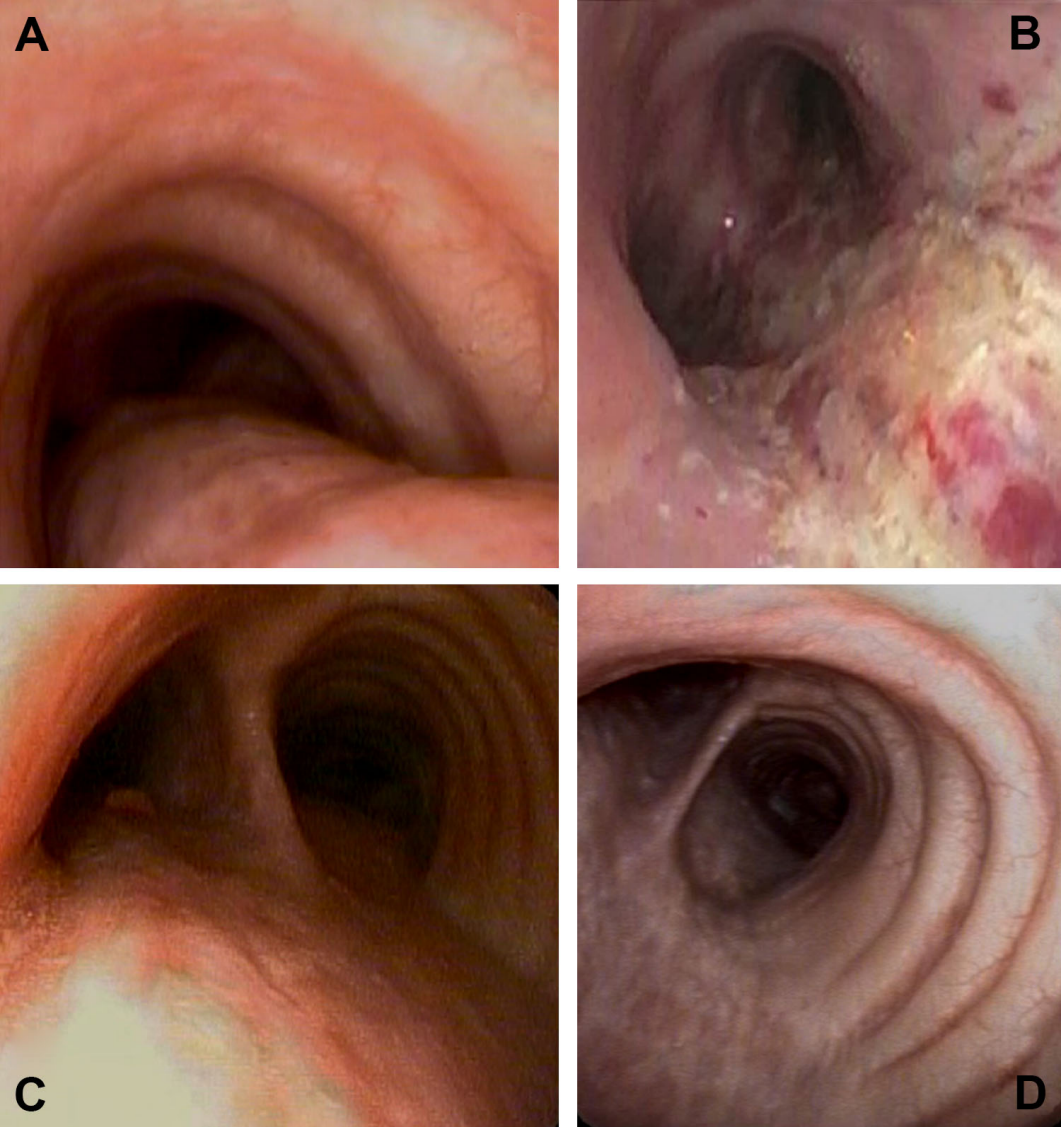
Endoscopic aspects of the trachea. **a** Preoperative collapse of the lower trachea and main stem bronchi during full exhalation. **b** Intraoperative view just after laser strafing of the mucosa of the posterior wall of the lower trachea and main stem bronchi. **c** Endoscopic view 2 weeks following laser tracheobronchoplasty showing granulation tissue. **d** Endoscopic view 12 weeks following the third stage of laser tracheobronchoplasty. Note that the posterior wall shows stiff fibrous tissue that does not collapse during full exhalation

The procedure is performed under general anesthesia. Perioperative systemic steroids of 125 mg of IV methylprednisolone sodium succinate (Solumedrol^®^, Pfizer Pharmaceutical, NYC, USA) and broad-spectrum intravenous antibiotics are administered. An oral endotracheal tube of an appropriate size is placed. A suspension laryngoscopy is performed using a Lindhölm^®^ laryngoscope system (#8587 A, KARL STORZ, Germany). The endotracheal tube is replaced by an 8.5 Mallinckrodt™ cuffed endotracheal tube (Covidien, Mansfield, MA, USA) and is placed within the subglottis with the cuff inflated at the level of the glottis and supraglottis to get access to the whole length of the trachea. The endotracheal tube is taped to the laryngoscope. A bronchoscopy swivel adapter is attached to the endotracheal tube. A high-resolution video bronchoscope (BF-1T180, Olympus, Tokyo, Japan) is introduced through the adapter. The FiO_2_ is gradually turned down to ≤0.40 to reduce the risk of airway fire during laser use. The holmium laser of the VersaPulse^®^ PowerSuite™ Dual Wavelength (Lumenis Ltd, Israel) is used at 40 pulses/s with 0.4 J per pulse and an average power density of 16 watts. The laser is delivered via a quartz fiber with a diameter of 273 μm (HLF-S273-SMA, Cook Ireland Ltd., Limerick, Ireland) which is introduced through the operating channel of the bronchoscope. The laser is used to strafe the mucosa of the posterior wall of the tracheobronchial tree longitudinally, transversely, and in a serpentine manner from distal to proximal. The fiber is pushed gently into the plane of the mucosa as the scope is withdrawn preventing uncontrolled deep laser penetration that could produce a pneumothorax or, worse, a great vessel injury (video clip). This technique produces relatively deep furrows into the submucosa while still preserving areas of intact mucosa between the strafes (Fig. 1). This is felt to increase wound healing while still promoting scarring to stiffen the mucosa. At the end of each mainstem treatment and at the end of the tracheal treatment, copious saline irrigation is performed to cleanse any blood and soft tissue debris from within the airway. No case required suction cautery to achieve hemostasis. On rare occasions, the YAG wavelength was used to cauterize small bleeders. The time length of the procedure is about 45 min to treat the whole trachea and the main stem, lobar, and segmental bronchi. Patients require between 2 and 3 laser tracheobronchoplasty procedures to stiffen their posterior tracheobronchial wall sufficiently. Approximately, a twelve week interval separates each stage from the next (Fig. 2).

In patients with concomitant tracheal stenosis, the scarred area was laser lysed (usually with the holmium laser), dilated with a semi-compliant 18 mm balloon (#M00558370 CRE, Boston Scientific, Boston USA) and injected with methylprednisolone (40 mg/cc suspension; Depo Medrol, Pfiser, USA).

### Postoperative care

Patients are admitted for airway observation for a minimum of 48 h in a monitored setting. They also get respiratory care including nebulized 3 % sodium chloride to help clear secretions (4 cc every 4 h and as needed for problems clearing secretions) along with broad spectrum IV antibiotics. Some patients are also given 20 % *N*-acetyl-l-cystine (Mucomyst^®^; Bristol-Myers Squibb Co., NYC, USA) along with ½ strength albuterol sulfate as an extra measure to clear reactive secretions and sloughing tissue/eschar. They are sent home with prescriptions for oral antibiotics for a week such as trimethoprim/sulfamethoxizole (Bactrim DS^®^, Roche, Basel, Switzerland) or ciprofloxacin (Cipro^®^ Bayer, USA) and a nebulizer to continue their 3 % hypertonic saline twice per day and as needed (with 20 % *N*-acetyl-l-cystine if needed). After discharge, they are followed-up in the outpatient clinic at 2, 6, and 12 weeks postoperatively. All patients had follow-up greater than 6 months after their last laser treatment.

## Results

The preoperative mean patient dyspnea score was 35.7 (range 32–40) and post operatively it was 12.5 (range 9–19). All cases showed significant improvement of their respiratory symptoms; the preoperative to postoperative difference was 23.3 (*P* < 0.001 with the 95 % confidence intervals of 20.38–26.02). The mean number of procedures was 2.3 per patient with the average laser energy delivered per procedure of ~1600 J. The decision to proceed with additional treatments was based on the endoscopic appearance of the tracheal bronchial tree and the patient report of breathing comfort. In seven cases the scale of dyspnea reported was so much improved after the second laser as to obviate the need for a third treatment. None to date have desired or required any additional treatment. No significant complications occurred in this series of patients. On interval bronchoscopies after surgery, most had small tufts of granulation tissue that were easily suctioned away by the bronchoscope. No cases of cicatricial scarring in the trachea or mainstem were encountered.

## Discussion

TBM has been reported to be present in up to 12 % of all patients undergoing bronchoscopy and in as many as 44 % of patients undergoing bronchoscopy who have a history of chronic bronchitis [5, 9]. TBM can be localized or diffuse. Localized TBM is usually seen in patients with prolonged endotracheal intubation or tracheostomy. Diffuse TBM is frequently seen in patients with emphysema and chronic bronchitis. TBM is a pathologic weakening of the airway that produces dynamic outflow obstruction with nonspecific symptoms such as dyspnea, orthopnea, intractable cough, and the inability to clear secretions, predisposing the patient to recurrent infections. Up to 7 % of patients with severe, diffuse TBM may present with respiratory failure requiring mechanical ventilation [16]. Others may have unexplained decannulation or extubation failure [16–18].

Multiple comorbidities have been associated with TBM including GERD, morbid obesity, and OSA. Obesity results in several anatomic changes, including diaphragm elevation and downward movement of the chest wall, which result in foreshortening and reduced rigidity of the trachea [19]. Repeated exposure to acid and digestive enzymes from GERD might alter the matrix protein structure, resulting in reduced tensile strength of the tracheal tissue, leading to TBM [17, 20]. Our study confirms this as TBM was associated with all of these. We stress the point that, in addition to surgical treatment of the TBM, obese patients should be encouraged to lose weight; and if GERD and aspiration cannot be controlled medically, surgical intervention such as with a laparoscopic Nissen fundoplication may be necessary. In addition, OSA should be treated with noninvasive positive pressure ventilation.

In spite of advances in radiologic imaging, our study supports the view that dynamic flexible bronchoscopy should appropriately be regarded as the gold standard for diagnosing TBM and guiding treatment. It permits real-time examination of the airways and provides precise information on morphology, extent, and location of pathology [21]. This has been confirmed in our study with all ten patients having greater than 50 % collapse and most fully collapsing on end-expiration. Only three cases of our patients showed airway collapse detected on the dynamic CT scan of the chest and the remaining seven cases were solely diagnosed on dynamic flexible bronchoscopy. Moreover, PFTs seem unreliable for the diagnosis of TBM and results that are within normal limit should not dissuade the clinician from pursuing this diagnosis [3, 22, 23].

Medical lasers are widely used in interventional pulmonology [24]. To our knowledge, no trial has been performed to assess the use of lasers in the treatment of TBM. In one case published in 2011 by Dutau et al., a patient with Mounier-Kuhn syndrome was successfully managed by treating the posterior collapse of the central airway with yttrium aluminum pevroskyte (YAG) laser at low power settings and using a rigid bronchoscope [25]. This condition, otherwise known as “megatrachea”, is rare and distinct from the much more common condition of TBM. The reported case underwent a second stage laser treatment 14 months later due to a recurrence of the posterior mucosal collapse. In our study, we used the holmium laser because it is highly absorbed by hemoglobin, producing wounds with excellent hemostasis. It also has a shallow depth of penetration to approximately 0.4 mm. The depth minimizes collateral damage to the surrounding tissues [26]. Based on our study, the holmium laser triggers a retractile fibrotic process that stiffens the posterior mucosal membrane with acute improvement in symptoms, and produces complete healing after 12 weeks. Additional stiffening of the posterior tracheobronchial wall is obtained by repeating the procedure once or twice. Despite our experience with rigid bronchoscopy, we have chosen to use flexible bronchoscopy to deliver the laser energy with reliable access to lobar and segmental bronchi thus targeting with high precision the malacic segments of the tracheobronchial tree. In addition, the use of a flexible bronchoscope enables our technique to be performed by clinicians who may not be familiar with rigid bronchoscopy. This preliminary report of our novel technique showed 100 % success rate in the treatment of TBM without major complications. We think that this technique could significantly impact the management of patients with TBM and look forward to performing prospective, multicenter studies.

## Conclusion

Laser tracheobronchoplasty is a novel, safe, easy to adopt, and effective technique for the treatment of TBM. It presents a simpler, safer alternative to open trans-thoracotomy tracheobronchoplasty and a durable alternative to endoluminal stenting.

## Electronic supplementary material

Below is the link to the electronic supplementary material.

Supplementary material 1 (MP4 16796 kb)
